# Supplementary material for: CNVs with adaptive potential in Rangifer tarandus: genome architecture and new annotated assembly
Source: Life Sci Alliance. 2021 Dec 15;5(3):e202101207. doi: 10.26508/lsa.202101207 (PMC8711850; doi:10.26508/lsa.202101207)
Supplement: Reviewer comments [file LSA-2021-01207_review_history.pdf]

Life Science Alliance

CNVs with adaptive potential in Rangifer tarandus: genome architecture and new annotated assembly

Julien Prunier, Alexandra Carrier, Isabelle Gilbert, William Poisson, Vicky Albert, Joelle Taillon, Vincent Bourret, Steeve Cote, Arnaud Droit, and Claude Robert
DOI: https://doi.org/10.26508/lsa.202101207

Corresponding author(s): Julien Prunier, Université Laval and Claude Robert, Université Laval

Review Timeline:

Submission Date:	2021-08-23
Editorial Decision:	2021-09-23
Revision Received:	2021-10-29
Editorial Decision:	2021-11-10
Revision Received:	2021-11-29
Accepted:	2021-11-29

Scientific Editor: Novella Guidi

Transaction Report:

(Note: With the exception of the correction of typographical or spelling errors that could be a source of ambiguity, letters and reports are not edited. The original formatting of letters and referee reports may not be reflected in this compilation.)

September 23, 2021

Re: Life Science Alliance manuscript #LSA-2021-01207-T

Julien Prunier
Genomic center - Laval University

Dear Dr. Prunier,

Thank you for submitting your manuscript entitled "Copy number variations with adaptive potential in caribou (Rangifer tarandus): genome architecture and new annotated genome assembly" to Life Science Alliance. The manuscript was assessed by an expert reviewer, whose comments are appended to this letter. We invite you to submit a revised manuscript addressing the Reviewer's comments.

To upload the revised version of your manuscript, please log in to your account: https://lsa.msubmit.net/cgi-bin/main.plex

You will be guided to complete the submission of your revised manuscript and to fill in all necessary information. Please get in touch in case you do not know or remember your login name.

While you are revising your manuscript, please also attend to the below editorial points to help expedite the publication of your manuscript. Please direct any editorial questions to the journal office.

The typical timeframe for revisions is three months. Please note that papers are generally considered through only one revision cycle, so strong support from the referees on the revised version is needed for acceptance.

When submitting the revision, please include a letter addressing the reviewer's comments point by point.

We hope that the comments below will prove constructive as your work progresses.

Thank you for this interesting contribution to Life Science Alliance. We are looking forward to receiving your revised manuscript.

Sincerely,

Novella Guidi, PhD
Scientific Editor
Life Science Alliance

A. THESE ITEMS ARE REQUIRED FOR REVISIONS

-- A letter addressing the reviewers' comments point by point.

-- An editable version of the final text (.DOC or .DOCX) is needed for copyediting (no PDFs).

-- High-resolution figure, supplementary figure and video files uploaded as individual files: See our detailed guidelines for preparing your production-ready images, https://www.life-science-alliance.org/authors

-- Summary blurb (enter in submission system): A short text summarizing in a single sentence the study (max. 200 characters including spaces). This text is used in conjunction with the titles of papers, hence should be informative and complementary to the title and running title. It should describe the context and significance of the findings for a general readership; it should be written in the present tense and refer to the work in the third person. Author names should not be mentioned.

-- By submitting a revision, you attest that you are aware of our payment policies found here: https://www.life-science-alliance.org/copyright-license-fee

B. MANUSCRIPT ORGANIZATION AND FORMATTING:

Full guidelines are available on our Instructions for Authors page, https://www.life-science-alliance.org/authors

We encourage our authors to provide original source data, particularly uncropped/-processed electrophoretic blots and

spreadsheets for the main figures of the manuscript. If you would like to add source data, we would welcome one PDF/Excel-file per figure for this information. These files will be linked online as supplementary "Source Data" files.

IMPORTANT: It is Life Science Alliance policy that if requested, original data images must be made available. Failure to provide original images upon request will result in unavoidable delays in publication. Please ensure that you have access to all original microscopy and blot data images before submitting your revision.

Reviewer #1 (Comments to the Authors (Required)):

In this manucript, Prunier et al., investigated genome architecture, especially copy number variation (CNV), among diverse populations of wild caribou (a vulnerable circumpolar species) in Canada. To achieve this goal, the authors greatly improved the genome assembly of the Caribou as well as annotation by combining very recent NGS technologies (i.e. long-read sequencing and linked-read seqeuencing). Thanks to this large sequencing effort, they provide a useful resource of copy number variant for this species. Finally, they investigated putative genetic adaptation by testing the relationship between CNVs and populations ecotypes.

In the last decade, the study of CNVs has largely been limited to specific genes of interest and to model organisms. Few studies to date have examined the role of CNVs in non-model species, despite recent analytical advances that allow their detection. This paper demonstrates that broad surveys of CNVs can bring substantial information about genetic variability among wild ecotypes and the inform about the role of structural variants in local adaptation.

I think the manuscript is well written, is easy to follow and it will make a very valuable contribution to structural variant resources in wild species. I think it's very close to being ready for publication, but I would suggest the following small additions/corrections:

[1] Introduction :

I enjoyed the reading of the introduction. I think that you can also mentionned that genome-wide CNVs datasets can also be used for population structure analyses, and that sometime, CNVs can support more genetic structure than SNPs (see Sudmant et al., 2015 and Dorant et al., 2020).

[2] Results :
In section Genome annotation inferred from RNAseq de novo assembly, second paragraph : « Transcripts were more numerous in the Transabyss transcriptome assembly (1,711,588) than in a5 one (223,597). This process resulted in the identification of 17,172 annotated genes based on the a5 assembly and 30,731 based on the Transabyss assembly. Overlap between both assemblies resulted in 20,419 corroborated annotated gene structures that were distributed over 2,759 genome assembly scaffolds. »
I'm not sure to fully understand how the overlap between 17,172 genes and 30,731 genes can result in 20,419 genes. Please, can you reword this sentence to clarify this result.

In section Large CNVs clustered in hotspots and encompassed coding sequences, end of the paragraph 1 : « CNVs were not randomly distributed over the genome assembly but clustered into 31 hotspots including 227 CNVs (KS test; D = 0.047 and p = 0.001; Fig. 5). »
This result is very interesting and I'm wondering if the authors also noted that many hotspots are localized at the scaffolds' boundaries (well visible in the yellow distribution). Does it there an explanation to this ? Could be a link between CNVs hotspot and the chromosomal position of these ? I think that it could be interesting to test if the hotspot position of scaffolds' boundaries is "random" or not with a statistical test.
This reflexion come from another study focused on CNVs in balsam poplar (Prunier al., 2018).

I noted that the authors performed a DAPC to identify putative CNVs loci related to caribou ecotypes (i.e. boreal desentary and migrating ecotypes). However, I did not find any information about the DAPC procedure itself. Did the authors used a DAPC with prior information ? Did they used a cross-validation information to select the number of substantial PCs ? How many PCs they used ? I think that this information should be added to the supplementary material.

On another side, to assess putative association between CNVs data and populations ecotypes, I would suggest to test it with a Redundancy Analysis (RDA), which is specifically built to test correlation between genetic and ecological data. Moreover, RDA allow users to define an FDR threshold to identify canditate loci involved in genetic adaptation. It would be easy and not so long (one month?) to compare the CNVs matrix with ecotype vector in such analysis.
Here is the well written tutorial available online at : https://popgen.nescent.org/2018-03-27_RDA_GEA.html

[3] Discussion

The discussion is well written and the authors greatly argue about possible bias or lack of data considering their dataset and their interpretations.

In the section « CNVs signatures related to adaptation in wild mammal ecotypes », I would suggest the study conducted by Rinker et al. (2019), which assess CNVs and adaptation in another circumpolar mammal species (i.e. bears). It could be possible to find interesting information in term of ecological adaptation. Note that this is just a litterature suggestion.

[4] Material and methods :
In part « Whole genome long-read sequencing » and « Transcriptome analyses », please add number of samples used for each approach.

In part « Whole-genome short-read sequencing of the various ecotypes », the number of samples used and the sex info have been indicated. Perfect !

Can the authors add a small part about identification of putative adaptive CNVs.

References cited :

Y. Dorant, et al., Copy number variants outperform SNPs to reveal genotype-temperature association in a marine species. Molecular Ecology, 4765-4782 (2020).
J. Prunier, et al., Gene copy number variations involved in balsam poplar (Populus balsamifera L.) adaptive variations. Molecular Ecology 28, 1476-1490 (2019).
D. C. Rinker, N. K. Specian, S. Zhao, J. G. Gibbons, Polar bear evolution is marked by rapid changes in gene copy number in response to dietary shift. Proceedings of the National Academy of Sciences 116, 13446-13451 (2019).
P. H. Sudmant, et al., Global diversity, population stratification, and selection of human copy-number variation. Science 349 (2015).

Dear editor,

We thank the anonymous reviewer for interesting insights and suggestions. We addressed all reviewer's comments and corrected the manuscript accordingly. You will see here after our detailed responses to reviewer but the main improvements and modifications are:

- Integration of all publications suggested by the reviewer in both introduction and discussion which substantially improved the manuscript.
- Clarification of the numbers for transcriptome assembly
- Addition of details regarding DAPC analysis and identification of putative adaptive CNVs in the "Material&Methods" section.

We hope this new version of the manuscript will meet your expectations.

Best Regards,

Julien Prunier, Claude Robert, on behalf of all co-authors.

Reviewer:
I think the manuscript is well written, is easy to follow and it will make a very valuable contribution to structural variant resources in wild species. I think it's very close to being ready for publication, but I would suggest the following small additions/corrections:

[1] Introduction :

I enjoyed the reading of the introduction. I think that you can also mentionned that genome-wide CNVs datasets can also be used for population structure analyses, and that sometime, CNVs can support more genetic structure than SNPs (see Sudmant et al., 2015 and Dorant et al., 2020).

Answer:
We agree and this is now mentioned in the first paragraph of the introduction section.

Reviewer:
[2] Results :
In section Genome annotation inferred from RNAseq de novo assembly, second paragraph : « Transcripts were more numerous in the Transabyss transcriptome assembly (1,711,588) than in a5 one (223,597). This process resulted in the identification of 17,172 annotated genes based on the a5 assembly and 30,731 based on the Transabyss assembly. Overlap between both

assemblies resulted in 20,419 corroborated annotated gene structures that were distributed over 2,759 genome assembly scaffolds. »
I'm not sure to fully understand how the overlap between 17,172 genes and 30,731 genes can result in 20,419 genes. Please, can you reword this sentence to clarify this result.

Answer:
We clarified the sentence by correcting the numbers. The number of annotated genes from the a5 and Transabyss assemblies were 20,419 and 30,731, respectively, while the final gff3 file includes 17,394 annotated gene models.
Reviewer:
In section Large CNVs clustered in hotspots and encompassed coding sequences, end of the paragraph 1 : « CNVs were not randomly distributed over the genome assembly but clustered into 31 hotspots including 227 CNVs (KS test; D = 0.047 and p = 0.001; Fig. 5). »
This result is very interesting and I'm wondering if the authors also noted that many hotspots are localized at the scaffolds' boundaries (well visible in the yellow distribution). Does it there an explanation to this ? Could be a link between CNVs hotspot and the chromosomal position of these ? I think that it could be interesting to test if the hotspot position of scaffolds' boundaries is "random" or not with a statistical test.
This reflexion come from another study focused on CNVs in balsam poplar (Prunier al., 2018).

Answer:
Indeed, hotspots appeared to be localized at scaffold boundaries. It is logical to assume that such boundaries are caused by the presence of repeated elements which hinder assembly. In turn, the presence of repeated elements which can be found locally or elsewhere in the genome can also be instrumental to the generation of CNVs, by favouring non-allelic homologous recombination for instance. However, we looked at the distribution of CNV hotspots positions upon a scaffold (taking into account scaffold length) following the reviewer’s comment and it was relatively homogenous.
Contrastingly to CNV work in balsam poplar (Prunier et al. 2018) where a genome assembly resolved at the chromosome level was available, our data does not permit testing for relative position regarding telomeres or centromeres.

Reviewer:
I noted that the authors performed a DAPC to identify putative CNVs loci related to caribou ecotypes (i.e. boreal desentary and migrating ecotypes). However, I did not find any information about the DAPC procedure itself. Did the authors used a DAPC with prior information ? Did they used a cross-validation information to select the number of substantial PCs ? How many PCs they used ? I think that this information should be added to the supplementary material.

Answer:
We applied the DAPC with the known ecotype for each individual as prior information. The number of substantial PCs was chosen according to the cumulative proportion of variance explained by PCs and accounted for 80% of the total variance. Only one discriminant function was retained to discriminate between both groups. This information was added in the “material

and methods” section of the new manuscript version (“CNV detection and characterization” subsection).

Reviewer:
On another side, to assess putative association between CNVs data and populations ecotypes, I would suggest to test it with a Redundancy Analysis (RDA), which is specifically built to test correlation between genetic and ecological data. Moreover, RDA allow users to define an FDR threshold to identify canditate loci involved in genetic adaptation. It would be easy and not so long (one month?) to compare the CNVs matrix with ecotype vector in such analysis.
Here is the well written tutorial available online at : https://popgen.nescent.org/2018-03-27_RDA_GEA.html

Answer:
Following reviewer’s suggestions, we looked into applying a Redundancy Analysis as described online (https://popgen.nescent.org/2018-03-27_RDA_GEA.html). This approach is an interesting alternative for Genotype-Environment Analysis (GEA) as it allows to delineate a combination of genetic markers and a combination of environmental factors presenting the maximum correlation. We found two limitations making the RDA not ideal in our case. First, the number of samples (N=19) spread into two groups (forest and migrant ecotypes) was somewhat limited to apply this kind of statistical analysis. The second objection is that we actually have only one environmental variable and moreover a categorical one, while a RDA is meant to summarize variation over several variables (mostly numerical ones as shown in the online workflow).
As a matter of fact, we wanted to apply something similar to GEA before choosing to apply a DAPC. When applying a DAPC, a categorical variable is expected to cluster individuals into groups and variants with a high loading scores upon a discriminant function between groups are the most varying amongst all variants. Thus a DAPC appears more adequate according to our sampling design. Similarly to what is described in the online tutorial for RDA, we selected outlier variants (outlier loading scores) as candidates to ecotype divergence.

Reviewer:
[3] Discussion
The discussion is well written and the authors greatly argue about possible bias or lack of data considering their dataset and their interpretations.

In the section « CNVs signatures related to adaptation in wild mammal ecotypes », I would suggest the study conducted by Rinker et al. (2019), which assess CNVs and adaptation in another circumpolar mammal species (i.e. bears). It could be possible to find interesting information in term of ecological adaptation. Note that this is just a litterature suggestion.

Answer:
As suggested, we referred to this publication in the mentioned section while exposing similarities in gene functions related to circumpolar adaptation in both genus despite the evolutionary distance between *Ursus* and *Rangifer*. Two interesting terms appeared in both

genus: molecular functions related to fatty acid metabolism which is known to mitigate hypothermia, and UV-response which is likely related to high latitude life.

Reviewer:
[4] Material and methods :
In part « Whole genome long-read sequencing » and « Transcriptome analyses », please add number of samples used for each approach.

In part « Whole-genome short-read sequencing of the various ecotypes », the number of samples used and the sex info have been indicated. Perfect !

Answer:
The whole genome long-read sequencing as well as transcriptome analyses were both performed from one female sample. This information was added in the "Material&Methods" section ("Whole genome long-read sequencing" subsection).

Can the authors add a small part about identification of putative adaptive CNVs.
Answer: As mentioned above, we added a few lines in the "material and methods" section ("CNV detection and characterization" subsection) to better describe the procedure we applied to identify putative adaptive CNVs.

References cited :

Y. Dorant, et al., Copy number variants outperform SNPs to reveal genotype-temperature association in a marine species. Molecular Ecology, 4765-4782 (2020).
J. Prunier, et al., Gene copy number variations involved in balsam poplar (Populus balsamifera L.) adaptive variations. Molecular Ecology 28, 1476-1490 (2019).
D. C. Rinker, N. K. Specian, S. Zhao, J. G. Gibbons, Polar bear evolution is marked by rapid changes in gene copy number in response to dietary shift. Proceedings of the National Academy of Sciences 116, 13446-13451 (2019).
P. H. Sudmant, et al., Global diversity, population stratification, and selection of human copy-number variation. Science 349 (2015).

November 10, 2021

RE: Life Science Alliance Manuscript #LSA-2021-01207-TR

Dr. Julien Prunier
Université Laval
molecular medecine
2705, boulevard Laurier
Québec, Quebec G1V 4G2
Canada

Dear Dr. Prunier,

Thank you for submitting your revised manuscript entitled "CNVs with adaptive potential in Rangifer tarandus: genome architecture and new annotated assembly". We would be happy to publish your paper in Life Science Alliance pending final revisions necessary to meet our formatting guidelines.

Along with points mentioned below, please tend to the following:

-please upload your main manuscript text as an editable doc file
-please upload your main and supplementary figures as single files
-please add the Twitter handle of your host institute/organization as well as your own or/and one of the authors in our system
-please consult our manuscript preparation guidelines https://www.life-science-alliance.org/manuscript-prep and make sure your manuscript sections are in the correct order and labeled correctly
-please upload your Tables in editable .doc or excel format
-please add your main, supplementary figure, and table legends to the main manuscript text after the references section
-please add an Author Contributions section to your main manuscript text
-please add a conflict of interest statement to your main manuscript text
-please add callouts for Figure 6A-B to your main manuscript text;
-please add a credit line for the photos in Figure 6

If you are planning a press release on your work, please inform us immediately to allow informing our production team and scheduling a release date.

LSA now encourages authors to provide a 30-60 second video where the study is briefly explained. We will use these videos on social media to promote the published paper and the presenting author (for examples, see https://twitter.com/LSAjournal/timelines/1437405065917124608). Corresponding or first-authors are welcome to submit the video. Please submit only one video per manuscript. The video can be emailed to contact@life-science-alliance.org

To upload the final version of your manuscript, please log in to your account: https://lsa.msubmit.net/cgi-bin/main.plex
You will be guided to complete the submission of your revised manuscript and to fill in all necessary information. Please get in touch in case you do not know or remember your login name.

To avoid unnecessary delays in the acceptance and publication of your paper, please read the following information carefully.

A. FINAL FILES:

These items are required for acceptance.

-- An editable version of the final text (.DOC or .DOCX) is needed for copyediting (no PDFs).

-- High-resolution figure, supplementary figure and video files uploaded as individual files: See our detailed guidelines for preparing your production-ready images, https://www.life-science-alliance.org/authors

-- Summary blurb (enter in submission system): A short text summarizing in a single sentence the study (max. 200 characters including spaces). This text is used in conjunction with the titles of papers, hence should be informative and complementary to the title. It should describe the context and significance of the findings for a general readership; it should be written in the present tense and refer to the work in the third person. Author names should not be mentioned.

B. MANUSCRIPT ORGANIZATION AND FORMATTING:

Full guidelines are available on our Instructions for Authors page, https://www.life-science-alliance.org/authors

We encourage our authors to provide original source data, particularly uncropped/-processed electrophoretic blots and spreadsheets for the main figures of the manuscript. If you would like to add source data, we would welcome one PDF/Excel-file per figure for this information. These files will be linked online as supplementary "Source Data" files.

Submission of a paper that does not conform to Life Science Alliance guidelines will delay the acceptance of your manuscript.

It is Life Science Alliance policy that if requested, original data images must be made available to the editors. Failure to provide original images upon request will result in unavoidable delays in publication. Please ensure that you have access to all original data images prior to final submission.

The license to publish form must be signed before your manuscript can be sent to production. A link to the electronic license to publish form will be sent to the corresponding author only. Please take a moment to check your funder requirements.

Reviews, decision letters, and point-by-point responses associated with peer-review at Life Science Alliance will be published online, alongside the manuscript. If you do want to opt out of having the reviewer reports and your point-by-point responses displayed, please let us know immediately.

Thank you for your attention to these final processing requirements. Please revise and format the manuscript and upload materials within 7 days.

Thank you for this interesting contribution, we look forward to publishing your paper in Life Science Alliance.

Sincerely,

Novella Guidi, PhD
Scientific Editor
Life Science Alliance

November 29, 2021

RE: Life Science Alliance Manuscript #LSA-2021-01207-TRR

Dr. Julien Prunier
Université Laval
molecular medecine
2705, boulevard Laurier
Québec, Quebec G1V 4G2
Canada

Dear Dr. Prunier,

Thank you for submitting your Research Article entitled "CNVs with adaptive potential in Rangifer tarandus: genome architecture and new annotated assembly". It is a pleasure to let you know that your manuscript is now accepted for publication in Life Science Alliance. Congratulations on this interesting work.

The final published version of your manuscript will be deposited by us to PubMed Central upon online publication.

Your manuscript will now progress through copyediting and proofing. It is journal policy that authors provide original data upon request.

Reviews, decision letters, and point-by-point responses associated with peer-review at Life Science Alliance will be published online, alongside the manuscript. If you do want to opt out of having the reviewer reports and your point-by-point responses displayed, please let us know immediately.

IMPORTANT: If you will be unreachable at any time, please provide us with the email address of an alternate author. Failure to respond to routine queries may lead to unavoidable delays in publication.

Scheduling details will be available from our production department. You will receive proofs shortly before the publication date. Only essential corrections can be made at the proof stage so if there are any minor final changes you wish to make to the manuscript, please let the journal office know now.

DISTRIBUTION OF MATERIALS:
Authors are required to distribute freely any materials used in experiments published in Life Science Alliance. Authors are encouraged to deposit materials used in their studies to the appropriate repositories for distribution to researchers.

You can contact the journal office with any questions, contact@life-science-alliance.org

Again, congratulations on a very nice paper. I hope you found the review process to be constructive and are pleased with how the manuscript was handled editorially. We look forward to future exciting submissions from your lab.

Sincerely,

Novella Guidi, PhD
Scientific Editor
Life Science Alliance
